# First Evidence of Spillover of *Rocahepevirus ratti* Into Humans in Thailand

**DOI:** 10.1155/tbed/9954682

**Published:** 2025-09-24

**Authors:** Julie Yamaguchi, Gregory S. Orf, Lester J. Perez, Pakpoom Phoompoung, Srisakul Chirakarnjanakorn, Yupin Suputtamongkol, Gavin A. Cloherty, Michael G. Berg

**Affiliations:** ^1^Infectious Disease Research, Abbott Laboratories, Abbott Park, Illinois, USA; ^2^Abbott Pandemic Defense Coalition, Abbott Laboratories, Abbott Park, Illinois, USA; ^3^Faculty of Medicine, Siriraj Hospital, Mahidol University, Bangkok, Thailand

**Keywords:** next-generation sequencing, Orthohepevirus C, rat hepatitis E virus, *Rocahepevirus ratti*, Thailand, virus discovery

## Abstract

*Rocahepevirus ratti* (hepatitis E virus [HEV]-C), originally discovered in rats in 2010, has been recently linked to hepatitis in humans. Although rare and typically detected in the immunocompromised, much like *Paslahepevirus balayani* (HEV-A), it can manifest as acute or persistent hepatitis. In a next-generation sequencing (NGS)-based screen for causes of acute febrile illness (AFI) in Thailand, we assembled a complete *Rocahepevirus* (rat HEV [rHEV]) genome from a patient admitted to the hospital who developed abnormal liver functions 2–3 months after a heart transplant. Despite withdrawal of medications suspected of inducing hepatitis, he progressed from parenchymal liver disease to cirrhosis. The absence of other viral etiologies suggested rHEV may have been the cause of chronic hepatitis. Thailand strain Ma617-09869 is the sole human representative in a clade of genogroup C1 composed of sequences found in rats from Thailand and neighboring Southeast Asian countries, including Laos, Cambodia, Vietnam, and Indonesia. Principal component analysis (PCA) of viral sequences indicates humans are incidental hosts and suggests that white bellied rats (*Niviventer* spp.) are the putative original host, with black and common rats (*Rattus* spp.) serving as the natural reservoir. While *Rocahepevirus* adaptation may not currently facilitate human-to-human transmission, specific diagnostics are needed to identify additional sequences and cases, not only to gain a better understanding of the biology of this virus, but also to assess the risk for continued evolution, virulence, and increased zoonotic events.

## 1. Introduction

Acute, food- or water-borne infections due to hepatitis A virus (HAV) and hepatitis E virus (HEV) are usually self-limiting but represent a significant burden of disease worldwide, with 1.5 and 20 million cases, respectively, annually [1]. Most strains of HEV infecting humans belong to the species *Paslahepevirus balayani* (previously referred to as *Orthohepevirus A*; HEV-A), which consists of eight genotypes [2]. Genotypes 1–2 result from fecal–oral transmission due to poor sanitation and hygiene, whereas genotypes 3 and 4 are more widespread geographically and result from close contact with, or consuming uncooked meat of, infected animals such as swine. The *Hepeviridae* family also includes genera restricted to bird (*Avihepevirus*), rat (*Rocahepevirus*), and bat (*Chirohepevirus*) hosts, formerly HEV-B/C/D, respectively [3]. *Rocahepevirus* (rat HEV [rHEV]) was discovered in rats in 2010 in Germany [4], then later detected throughout Europe [5] and more broadly worldwide (see [6] and references therein). Related viruses have also been detected in voles, shrews, foxes, ferrets, and other mammals [7–10]. Though originally believed to be restricted to rodents and lower mammals, *Rocahepevirus* was first detected in humans in 2018 in a liver transplant patient from Hong Kong with chronic hepatitis [11]. The authors went on to identify seven more cases in Hong Kong [12], and since then, most human infections (16/21; 76%), and rodent rHEV sequences (586/817; 71%) have been found in Asia [6, 13, 14]. Experiments in human cell culture suggest that virus shed in human fecal samples leads to abortive infections, whereas virus derived directly from rat liver homogenate still replicated efficiently in several human liver cell lines [11, 15, 16]. Against the backdrop of detection in a variety of sample types [11] in multiple patient cases from different countries [6, 10], the consensus is that this is indeed a virus with clear zoonotic potential and a cause of hepatitis [17].

The clinical picture that has emerged is that immunosuppressed individuals (solid organ transplant recipients, cancer patients, those with underlying chronic disease such as diabetes or hypertension) are at greatest risk of infection [6]. Nevertheless, immunocompetent people are still susceptible, as evidenced by a previously healthy Canadian individual contracting the virus while working for the United Nations in Central Africa (DRC or Gabon) [18] and two previously healthy individuals from Spain reporting acute hepatitis [19]. rHEV infection resembles that from HEV-A in terms of its acute and self-limiting nature, often accompanied by fever, fatigue, nausea, and abdominal pain, but it can also persist chronically or present sub-clinically [6]. Unfortunately, one of the seven cases from Hong Kong died of meningoencephalitis [12], and the third case in the Spanish cohort with metastatic oral cancer died of renal and liver failure [19], each potentially attributable to rHEV infection. Also, like HEV-A, rHEV is presumably transmitted through contaminated food or water, as well as through virus shedding in rat feces [11], but not their urine [20]. As all human cases have been detected in the last 6 years (2018–2024), and with estimates that up to 28% of rats in China [21] and 30% in Spain [22] carry the virus, this epidemiologic picture will continue to evolve as we learn more about this virus.

In the course of screening acute febrile illness (AFI) patients in Thailand, we detected rHEV in one of the patients by metagenomic sequencing (mNGS) and assembled the full genome. The patient's medical history and clinical presentation matched the profile for rHEV infection. We determined that this is the first recorded human case in Thailand. Furthermore, the genome clusters within a clade comprised of rat-hosted sequences from nearby Asian countries.

## 2. Materials and Methods

### 2.1. Study Cohort

Patients were enrolled in a retrospective viral discovery project to evaluate causes of unresolved acute undifferentiated fever in Thailand. The study was approved by the Siriraj Institutional Review Board committee (COA Si.391/2021). Serum or plasma was collected from leftover specimens and stored at a temperature of −70°C. Patient 09869 presented with a fever and an unknown cause of hepatitis. Consent for this case report was waived by the Review Board committee.

### 2.2. Extraction of Nucleic Acids and Preparation of Libraries for Next-Generation Sequencing (NGS)

Patient 09869 serum was treated with Benzonase (MilliporeSigma, Burlington, MA, USA) to deplete cell-free host nucleic acids, and then the remaining nucleic acids were extracted on a KingFisher Apex (ThermoFisher Scientific, Waltham, MA, USA). Metagenomic cDNA libraries were synthesized from total nucleic acids using SuperScriptIV and Sequenase v2.0 (ThermoFisher Scientific, Waltham, MA, USA) and barcoded using an Illumina Nextera XT v2 library prep kit (Illumina, Inc., San Diego, CA, USA) and unique dual index IDT-Nextera adapters (Integrated DNA Technologies, Coralville, IA, USA). Metagenomic (mNGS) libraries were sequenced by Azenta Life Sciences (Burlington, MA, USA).

### 2.3. Target Enrichment of mNGS Libraries

Sets of 24 mNGS libraries were pooled for enrichment of viral sequences using the Comprehensive Viral Research Panel (CVRP, Twist Biosciences, South San Francisco, CA, USA), composed of over 1M unique probes covering reference sequences for 3153 viruses, including 15,488 different strains, as previously described [23, 24]. CVRP target-enriched (CVRPte) libraries were sequenced on an Illumina NextSeq 1000 instrument. NGS fastq files were processed to remove low-quality and human sequences and to identify remaining microbial reads using DiVir 3.0, an in-house metagenomics and virus discovery pipeline.

### 2.4. RT-PCR and SMART-PCR to Fill Gaps Remaining in the Genome After mNGS and CVRP teNGS

Two approaches were used to fill three large gaps in the genome following NGS. Standard RT-PCR1 and PCR2 amplification of gap 1 (G1-F1: CAGGGGCTWGAYAATTGGAC, G1-R1: ARCATCTCCAAAAGGACCCTCT, G1-F2: MTTACAGGCTGCYTRTTTGAGGC, G1-R2: CCGYGGRGGGTCARGGGCCA); gap 2 (G2-F1: CAGAGGGTCCTTTTGGAGAT, G2-R1: CGCATRACCTCCACATCATCA, G2-F2: TACCAGAYGGYGCCGCAGT, G2-R2: TCTCCTTRCCATACCTYCCCACA); and gap 3 (G3-F1: TCGGCTTGGTCTAAAACYCTTGT, G3-R1: TGGGCCACRATCCGCACAT, G3-F2: CCYTGTTTGGCCCTTGGTT, G3-R2: TGGACTGCGGCCAGAATGAC) were done at 50°C for 30 min, 98°C for 2 min, then 40 cycles of 98°C for 15 s, 58°C for 30 s, 72°C for 1 min, and 95°C for 10 min, then 40 cycles of 95°C for 15 s, 55°C for 30 s, and 72°C for 2 min, respectively, using the SuperScriptIV OneStep RT-PCR System (Invitrogen, Waltham, MA, USA) and AmpliTaq Gold DNA Polymerase (Applied Biosystems, Waltham, MA, USA). Sequences of all three fragments were obtained directly by Sanger sequencing on a 3130xL Genetic Analyzer (Applied Biosystems, Waltham, MA, USA) and edited using Sequencher PC Version 5.4.6 – Build 3767 software (Gene Codes Corp, Ann Arbor, MI, USA).

SMART-PCR libraries were prepared in parallel to the standard PCR fragments using the SMART PCR method described previously [25], a SMARTer PCR cDNA Synthesis Kit (Takara Bio USA, Inc., Mountain View, CA, USA), and rHEV reverse primers modified for SMART PCR (SMT-G1: AAGCAGTGGTATCAACGCAGAGTACARCATCTCCAAAAGGACCCTCT, SMT-G2: AAGCAGTGGTATCAACGCAGAGTACCGCATRACCTCCACATCATCA, SMT-G3: AAGCAGTGGTATCAACGCAGAGTACTGGGCCACRATCCGCACAT,

SMT-G4: AAGCAGTGGTATCAACGCAGAGTACAGAAAAACTAGACACTGTCGG). This approach is ideal for filling gaps and resolving genome termini, as it combines 5′ RACE with NGS to enable cDNA synthesis of transcripts with defined 3′ but variable 5′ ends. cDNA libraries were amplified using primers complementary to SMART tags affixed to the ends of the inserts.

The PCR fragments and SMART PCR libraries were barcoded using both an Illumina Nextera XT v2 library prep kit with IDT-Nextera adapters and a SparQ DNA Frag and Library Prep Kit (QuantaBio, Beverly, MA, USA) with QuantaBio-TruSeq adapters. Libraries were assessed, diluted, and loaded on a MiSeq System (Illumina) according to manufacturer recommendations. rHEV reads were mapped and assembled using the CLC Genomics Workbench (Qiagen Corp, Germantown, MD, USA).

### 2.5. Sequence Comparisons and Phylogenetic Tree Construction

All nucleotide sequences belonging to the family *Hepeviridae* (taxid: 3079366), excluding the genus *Piscihepevirus*, were retrieved from GenBank on May 20, 2024. Our fully assembled rHEV genome was then analyzed with an in-house implementation of BLAST, and the top 1000 hits from the nt database were retrieved; any that were absent from the taxonomy-based dataset (i.e., true *Hepeviridae* with incorrectly labeled taxonomy) were then included in the dataset. Completely identical sets of sequences were condensed to one representative (i.e., de-duplication), and all sequences <5000 nt in length were excluded. Thus, a comprehensive dataset containing our new rHEV genome and 1330 relatives from the *Hepeviridae* was constructed.

Due to the variable levels of coverage in the 5′ and 3′ untranslated regions in the dataset, the genomes were aligned using the L-INS-i algorithm implemented in MAFFT v.7.487 [26]. This allowed for confident identification of the 5′ and 3′ untranslated regions, which were then stripped from the alignment. Reconstruction of a maximum-likelihood phylogeny of this final alignment was achieved using IQ-TREE v.2.1.3 [27] essentially as described previously [24].

### 2.6. Principal Component Analysis (PCA)

To further interrogate the molecular sequences and uncover potential signatures linked to host-viral interactions, we conducted an in-depth analysis focusing on the relationships between different hosts identified for *Rocahepevirus ratti* genotype 1. All sequences from this species were considered for a PCA as delineated in Perez et al. [28]. Initially, an evolutionary distance matrix was calculated to determine the nucleotide divergence among the groups. This calculation was performed using the MEGA X software, employing the gamma Tamura–Nei model, which adjusts for rate variation among sites and accommodates varying substitution rates across nucleotide sites. The resulting distance matrix of nucleotide divergence served as the input for the PCA, which was conducted using the PCO software [29]. We elected not to transform or standardize the data further, maintaining the original scale differences for our analyses. For distance calculation, we employed the Bray–Curtis dissimilarity measure to account for compositional differences rather than mere presence or absence of traits. The analysis was conducted using discriminant analysis to find axes that maximize separation among the predefined groups of observations. Additionally, to assess the statistical significance of our findings, we conducted a permutation test involving 9999 random permutations. This robust method helps in evaluating the patterns observed against a null hypothesis of no association. A specific integer seed was used for these permutations to ensure the reproducibility of our results. The results themselves were plotted as a three-dimensional scatter plot using the plotly library for Python 3.

## 3. Results

**Figure 1:**
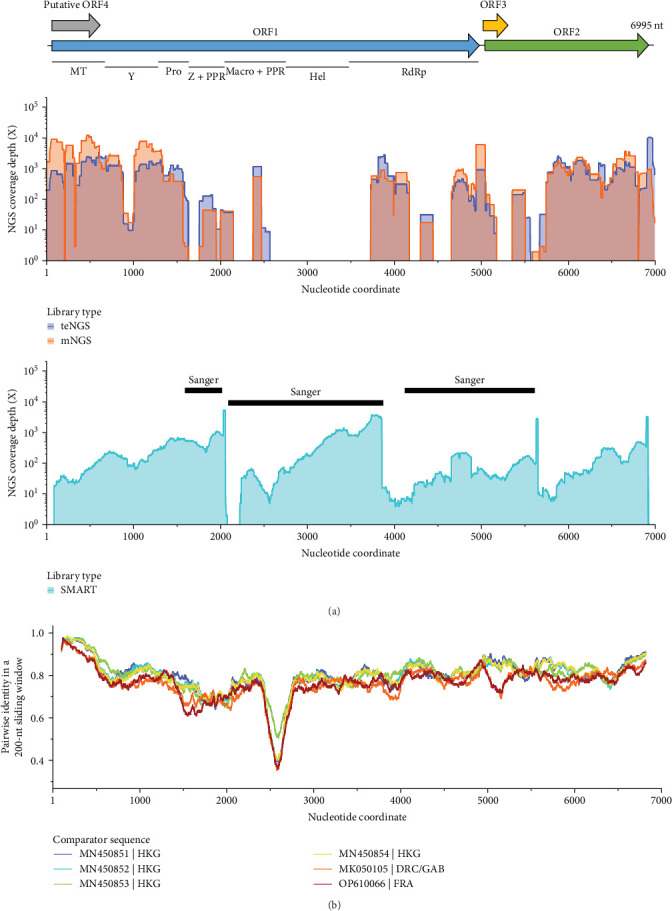
*Rocahepevirus* sequence assembly and similarity to reported strains: (A) genomic structure inferred from a 6995-nt contig assembled from patient 09869 using a combination of teNGS reads, mNGS reads, SMART-NGS reads, and Sanger sequencing of sub-genomic amplicons. (B) Genome-wide nucleotide pairwise comparison of the newly recovered sequence against other *Rocahepevirus* strains recently reported from human infections acquired in Asia, Europe, and Africa.

While exploring cases in Thailand with AFI by NGS, we detected the presence of reads corresponding to rodent HEV, or *Rocahepevirus* (rHEV), in patient 09869. An initial mapping and de novo assembly of reads from metagenomic (mNGS) and virus target-enriched (teNGS) libraries enabled us to compile approximately 65% of the putative genome. A total of 38,775 mNGS and 65,590 teNGS reads were obtained at 593X and 1221X depths, respectively. Using additional strategies, including RT-PCR with Sanger and SMART-PCR with NGS, the remaining gaps were filled in, and a nearly complete 6995 nt genome was assembled (accession PX021458; Figure 1A). A pairwise nucleotide comparison to other rHEV strains obtained from human subjects revealed that Ma617-09869 possessed approximately 80% identity across its genome to strains isolated in Hong Kong, Spain, and Germany (Figure 1B). We note, as have others [30], that the macro + PRR region in ORF1 (nt 2400–2700) is a highly variable region and may not share high homology, even in otherwise similar strains. A rare zoonosis with this level of sequence divergence indicated further investigation was warranted.

**Figure 2:**
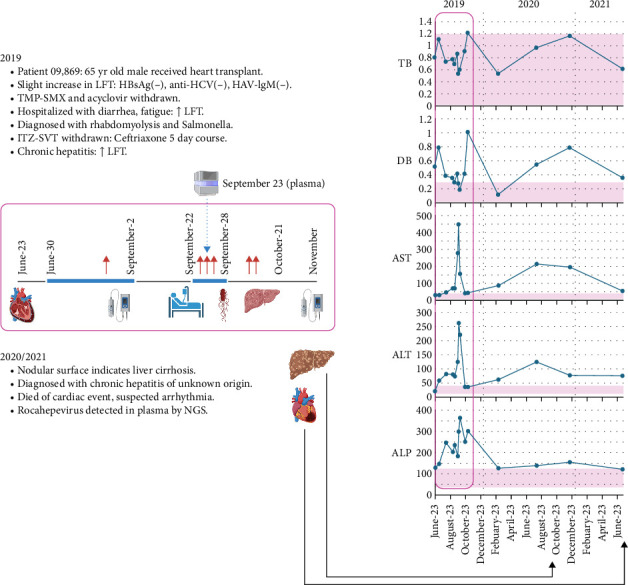
Clinical course for patient 09689 following heart transplantation. Magenta circles highlight the timeline of events and corresponding LFT results in 2019. Blue bar indicates the duration of trimethoprim/sulfamethoxazole and acyclovir administration, and the purple bar indicates itraconazole/simvastatin and ceftriaxone taken as an in-patient. Red arrows depict increases in liver function tests (pink intervals in LFT graphs indicate normal ranges; Table 1 for numeric values). The plasma sample sequenced was obtained the day after admission.

We first reviewed the clinical history to determine whether this individual was at risk for infection and whether the symptoms exhibited were consistent with what has been recently learned about rHEV (Figure 2). Patient 09869 was a 65-year-old male patient diagnosed with ischemic cardiomyopathy who underwent a heart transplantation on June 23, 2019. Preheart transplant evaluation results were: negative HBsAg, negative anti-HBc IgG, negative anti-HBs IgG, negative anti-HCV, positive CMV IgG, positive EBV IgG, negative HSV IgG, and positive VZV IgG, with no evidence of transaminitis (TB: 0.81 mg/dL, DB: 0.52 mg/dL, AST: 29 U/L, ALT: 21 U/L, ALP: 129 U/L), but this worsened 2 months post-transplant. Although the patient had a history of alcohol consumption for many years, he had abstained for 2 years prior to the heart transplant; he denied herbal use. Patient received itraconazole and simvastatin since transplantation: itraconazole 200 mg/day and simvastatin 10 mg/day. An abdominal ultrasound performed in August 2019 showed only a mild degree of parenchymal liver disease without nodular surface. Blood tests were all negative for HBs Ag, anti-HCV, and HAV-IgM. Therefore, he did not have alcoholic, or hepatitis B, or hepatitis C chronic liver disease. Trimethoprim/sulfamethoxazole and acyclovir, which were received since June 30, 2019, were discontinued on September 2, 2019, due to the assumption that his abnormal liver tests were attributed to drug-induced liver injuries.

He was admitted to Siriraj Hospital in Bangkok on September 22, 2019, due to watery diarrhea and fatigue lasting 6 days. His medications included prednisolone 10 mg/day, mycophenolate 1.5 g/day, tacrolimus 1 mg/day, itraconazole 200 mg/day, and simvastatin 10 mg/day. He denied alcohol drinking, herbal use, and eating uncooked food. Physical examination revealed that he was afebrile and mildly dehydrated. On the day of admission, his complete blood count levels were relatively normal: Hb: 12.9 g/dL, Hct: 39.1%, WBC: 6360 cells/mm^3^ (*N*: 83%, *L*: 3%, band: 8%), Plt: 154,000 cells/mm^3^. However, his elevated creatine levels (1.25 mg/dL) and abnormal liver function tests (TB: 0.87 mg/dL, DB: 0.42 mg/dL, AST: 278 U/L, ALT: 125 U/L, ALP: 184 U/L, Alb: 3.3 g/dL, Glo: 2.7 g/dL), which persisted for >1.5 weeks, suggested acute hepatitis (Table 1). Blood tests showed negative EBV IgM, negative HEV IgM, and positive HEV IgG. Urinalysis showed occult blood positive without red blood cells demonstrated in urine, which raised suspicion of rhabdomyolysis. Creatinine phosphokinase was 4785 U/L. Jaundice was not noted on initial follow-up examinations. Hemoculture yielded no growth, but *Salmonella* group G grew from stool culture. Immunofluorescence for *Leptospira spp*, *Rickettsia typhi*, and *Orientia tsusugamushi* were all negative. Patient 09869 was diagnosed with (1) *Salmonella* gastroenteritis and (2) drug-induced hepatitis with rhabdomyolysis (suspected interaction between itraconazole and simvastatin). He was treated with intravenous ceftriaxone for 5 days, and the culprit drugs were discontinued. Liver functions only slowly improved despite drug discontinuation, and he was discharged with oral antibiotics after 6 days of admission.

Renal function tests returned to baseline after 2 weeks of drug discontinuation. Despite improvement in liver function tests in October 2019 and withdrawal of all suspected culprit drugs, the patient continued to exhibit chronic active hepatitis (Table 1). A thorough re-evaluation of all medications uncovered no potential etiologic agents. A repeated liver ultrasound in November 2019 and October 2020 revealed heterogeneous echogenicity of the liver parenchyma with a mildly nodular surface, findings consistent with progressive liver cirrhosis. He was diagnosed with chronic hepatitis of unknown etiology, leading to liver cirrhosis, Child-Pugh class A. He died at home from a witnessed sudden cardiac death, ~2 years later, on August 7, 2021, suspected to be due to arrhythmia as assessed by his primary cardiologist. Sequenced plasma in which rHEV was detected was stored on September 23, 2019 (Figure 2). Considering that patient 09869 was likely immunocompromised due to heart transplantation and had chronic hepatitis with cirrhosis of unknown cause, these factors are compatible with the clinical spectrum of rHEV infection.

**Figure 3:**
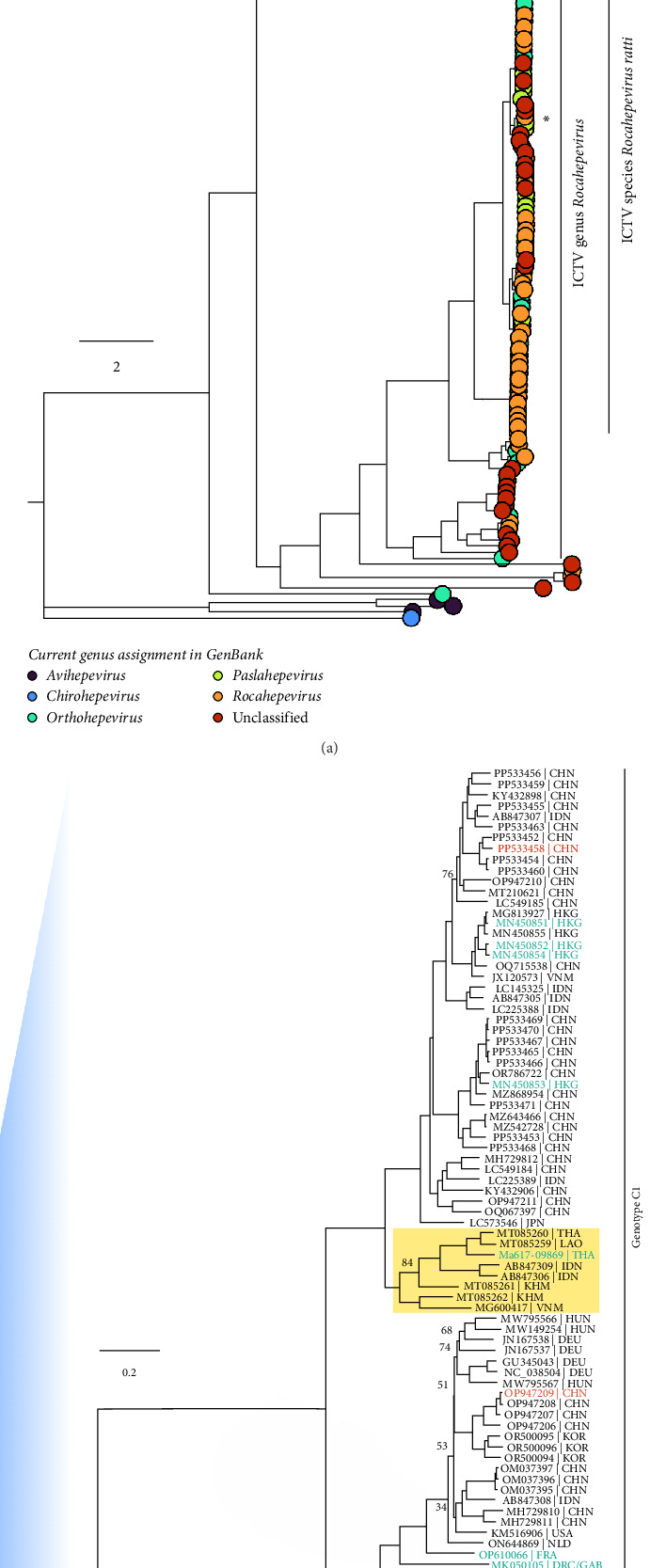
Taxonomic classification of the *Rocahepevirus* sequence from patient 09869: (A) maximum-likelihood phylogenetic tree of the Orthohepevirinae sub-family, with tips (taxa) colored based on the genus assigned in GenBank. The *Rocahepevirus* genus, defined by ICTV, has been expanded. The Ma617-09689 is denoted by the asterisk. (B) Maximum-likelihood phylogenetic sub-tree of panel A assigning two ICTV-defined genotypes in *Rocahepevirus ratti*. Ma617-09689 resides in the clade highlighted in the yellow box. Tips (taxa) are colored based on the taxonomic family of the host organism. Support values from ultrafast bootstrapping are shown at those nodes where support is less than 90%. Sp., species; Gt., genotype.

Next, to understand phylogenetically where this new sequence is positioned among Rocahepeviruses, we analyzed it in the context of previously reported strains in the *Orthohepevirinae* subfamily, which consists of *Paslahepevirus* (human, ungulates), *Avihepevirus* (avian), *Rocahepevirus* (rodent, mustelid), and *Chirohepevirus* (bat) genera (Figure 3A). Ma617-09869 clusters within the species *R. ratti* with a bootstrap >90%. Notably, numerous strains branching within the genus *Rocahepevirus* belong to a different genus according to their GenBank records, highlighting the need for updating the database to conform with ICTV classifications (Figure 3A). Two genotypes (C1–C2) have been defined within *R. ratti* [10] (Figure 3B). Genotype C2 is comprised entirely of sequences obtained from ferrets (Mustelids), whereas genotype C1 is a mixture of strains recovered from rats (Murids), shrews, and humans. The phylogenetic reconstruction of C1 indicated the formation of four major monophyletic clades (Figure 3B).

Patient Ma617-09869 appears to be the first human case in a clade where all the remaining sequences were obtained from rats (Figure 3B, yellow box). The closest related strains were isolated from red spiny rats (*Maxomys surifer*) from Laos (MT085259) and Thailand (MT085260). Also, within this clade were strains isolated from rats in Indonesia, Cambodia, and Vietnam. While rHEV strains from China are found in the other three clades, they are notably absent from this one.

**Figure 4:**
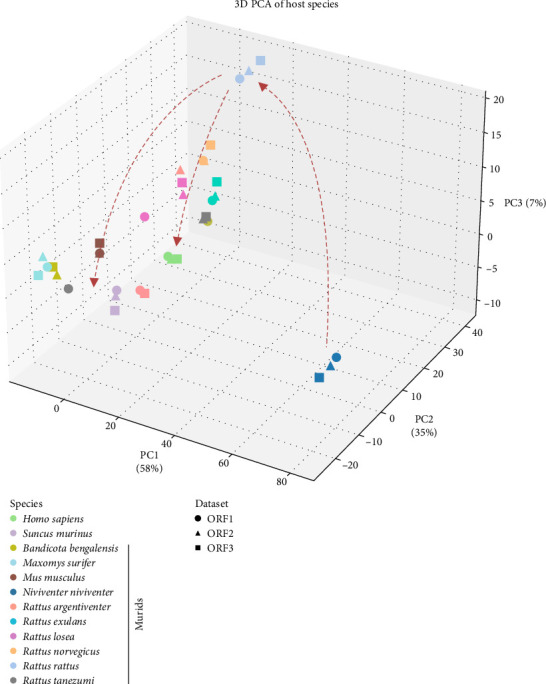
Principal component analysis (PCA) for ORF1-3 of *Rocahepevirus ratti* genotype 1 from the *Rocahepevirus* genus. The plot illustrates the segregation of sequences according to host species. The weighting of each principal component (PC1–PC3) in separating the host species is indicated on the axes. Arrows represent the inferred transmission pathways among the different hosts, highlighting potential routes for viral spread and interaction within the host population.

Inspection of multiple sequence alignments did not reveal any obvious amino acid changes that might explain the jump from rats to humans. As an alternate approach, we performed a PCA of nucleotide sequences to gauge the evolutionary distance of Ma617-09869 and other human sequences from those obtained in other host species. Applying the Temura–Nei substitution model, which relates evolutionary rates, transitions vs. transversions, etc., and grouping sequences according to host using the PCO software, we mapped ORFs 1-3 in three-dimensional space. The first observation was that each open reading frame from a given host clustered together, indicating that the unique molecular signature acquired within the host was shared throughout the genome and unlikely to be altered by recombination. Given the wide distance of *Niviventer niviventer* (white bellied rats) from the other hosts, we deduced that this species (found in India, Bhutan, and Pakistan) was likely the original host of *R. ratti*. *Rattus rattus* also appeared to be an outlier host but was more closely related to all the other species, including humans (Figure 4). This spatial arrangement suggests that domestic rats are the reservoir host and the remainder are, in fact, incidental hosts. Thus, whereas viruses in *Niviventer niviventer* and *Rattus rattus* stand apart as having acquired molecular signatures indicative of a coevolutionary process, the clustering of sequences together from humans and other hosts (i.e., various murids and shrews) (Figure 4) suggests a lack of molecular markers that could signify adaptation to these hosts. This pattern suggests that the viral jump to humans and other rodents from rats might be a relatively recent event, and the virus has not yet undergone sufficient adaptations in these new hosts to lead to a true speciation event. Given this context, the risk of human-to-human transmission appears low, and the outcome of infection (be it acute, chronic, or asymptomatic) is likely to vary widely based on host immune status (e.g., immunocompetent *versus* immunocompromised).

## 4. Discussion

*Rocahepevirus* is an emerging zoonosis, though at present it appears to be rare and primarily affects the immunocompromised [14]. Patient 09869, the first human rHEV infection described in Thailand, had received a heart transplant shortly before exhibiting signs of acute hepatitis, which eventually progressed to parenchymal liver disease and cirrhosis. Removal of medications potentially causing drug-induced liver injury did not ameliorate abnormal LFT values, nor were any common viral causes of hepatitis (e.g., HBV, HCV, HEV, HAV) detected; thus, a zoonotic or atypical infection like rHEV remains a plausible etiology. Transmission via solid organ transplant has proven a common route of exposure, with >25% of documented human infections attributed to liver and kidney transplants [6]. In addition to our patient and another subject from a Hong Kong cohort receiving a hematopoietic stem cell transplant (HSCT) [14], the individuals comprising these cases developed persistent hepatitis or cirrhosis. Chronic infections following solid transplants are also seen with HEV [31]; but by contrast, those experiencing acute hepatitis were either battling cancer, had an underlying comorbidity (e.g., hypertension, rheumatoid arthritis, diabetes), or were immunocompetent. It is reasonable to suggest, as others have [6], that both organ transplant recipients and blood transfusion recipients are potentially at risk.

The larger question is whether rHEV poses a danger to the broader population. Thus far, the majority of human cases have been detected in China, with 17 from Hong Kong, six from Yunnan, and one from Hainan [13, 14]. There have been several studies conducted in Germany, France, and Hungary in transplant/immunocompromised/acute hepatitis cohorts, but no positives have been detected [32, 33] until recently in Germany: 1 positive out of 3268 specimens tested [34]. Still, we have seen that otherwise healthy individuals are still susceptible and that infections can present sub-clinically, facilitating cryptic circulation. Indeed, even children are susceptible, with 2/11 subjects with acute hepatitis in Spain testing positive for rHEV: one was immunocompetent while the other had lymphoblastic lymphoma [35]. Seroprevalence estimates were performed in Hong Kong, comparing recipients of solid organ transplants (1.2%; 7/599) to immunocompetent matched controls (0.7%; 4/599) [36]. Similarly low incidence rates were found in Spain, with only 1.2% (2/169) of acute hepatitis of unknown etiology cases testing positive for rHEV [19]. Anti-rHEV IgG positivity rates are the same for people living with HIV (1.1%; 9/842) [37], although they are noticeably elevated (4.9%; 17/341) in homeless, injection drug users [38]. With estimates of prevalence in rats in Germany [39] and Spain [22] at 14.7%–41.2% and 30%, respectively, dense, urban areas prone to contamination of food or water by rodents provide the perfect setting for transmission, and those living in unhygienic conditions (e.g., the homeless) are at particular risk. Detection of rHEV RNA in pigs also raises the specter of livestock serving as intermediate hosts [40] and represents another threat to public health via pork consumption [41].

Ma617-09869 branches with rat-only sequences from Southeast Asia and shares ~80% nt identity with other human strains in genotype C1 (Figures 1B and 3B). According to the newly proposed distance-based sub-typing by Hon-Yin Lo et al. [42], this sequence belongs to rHEV clade II.c. Unlike its sister-clade, it currently lacks sequences from China, nor does it resemble the third cosmopolitan clade, which encompasses strains from Asia, Europe, Africa, North America, and South America. However, as more sequences are identified in the future, this composition is likely to change, and we will observe panmixia of isolates to indicate that geography neither imposes a delineation of clades nor restricts viral spread. What we can say definitively now, though, is that our study provides further evidence that genetically diverse *R. ratti* strains continue to jump to humans.

Our PCA indicated that *Niviventer* white bellied rats, native to central-east Asia, are the likely original hosts of rHEV (Figure 4). OP947208 was not evaluated in isolation, but rather in relation to the entire data structure: the PCA method is well-suited to reveal patterns and groupings that correspond to evolutionary relationships, adaptation strategies, or ecological associations between viruses and their hosts [43, 44]. Nevertheless, we acknowledge this conclusion is based on one available sequence and that a more robust dataset obtained from increased surveillance could change our interpretation. Given the limited number of sequences per host included in our PCA analysis, our results must be interpreted as associative links, since further modeling using coevolution/cospeciation dynamics and host-jump events as described [45] should be performed to better clarify rHEV host dynamics. The Asian musk shrew, *Suncus marinus*, has been suggested to be a reservoir for the virus [46], but in our study, it was grouped among other incidental hosts. Unfortunately, the presumed intermediate host, *Rattus rattus*, is distributed worldwide and is likely the reason that this virus has managed to spread to each continent. Sequences found in humans are highly related or nearly identical (99%) [11] to rodent strains collected in the same location, supporting the PCA data, which suggests these are spillovers into dead-end hosts and that person-to-person transmission is unlikely. However, as we have witnessed with countless other viruses (e.g., Zika virus, Chikungunya virus, SARS-CoV-2), accumulation of the right mutations could alter its adaptability to humans, leading to a variant with greater fitness, transmissibility, or virulence [47, 48].

The need for sensitive and specific diagnostics to have a true understanding of seroprevalence and to detect viremia has been stressed repeatedly [36]. Relying upon cross-reactivity or discordant results from commercial HEV antibody kits, whether convenient or the only available means, is an inadequate diagnostic approach for rHEV going forward. As mentioned, an ORF2 (capsid) peptide-based, enzymatic immunoassay (EIA) has been developed recently that distinguished HEV-A from HEV-C1 (rHEV) and arrived at an estimate of 0.92% IgG seroprevalence for the latter [36]. In parallel, the same group has identified pairs of capture and detection antibodies to build an antigen assay that distinguishes HEV-A from HEV-C1 infections [49]. High-throughput, fully automated platforms for both assays will be necessary for large-scale serosurveys or blood screening. For molecular detection, endpoint, gel-based PCR [50] is being replaced by quantitative RT-qPCR assays [11, 32]. We and others have unintentionally detected rHEV through unbiased metagenomics [51], though the viral load in our Thailand patient was undoubtedly low, given the challenges we faced assembling the genome. Unfortunately, the limited sample volume and nucleic acid hindered further molecular and serological studies. Hong Kong patient 5 (with a kidney transplant) had an infection for >1 year, an altered mental status, and eventually died, but measured only 10^3^ copies/ml in CSF at death [11]. These observations indicate that a sensitive, high-throughput, validated RT-qPCR comprehending the diversity of sequences will be necessary to ensure that active infections are not being underestimated. Ideally, awareness of this potential new cause of hepatitis will grow faster than the number of new cases, and diagnostics will keep pace to curtail its spread.

## Figures and Tables

**Table 1:** The following table shows abnormal liver function test results before and after admission.

Liver function test	Reference range	June 23, 2019	July 9, 2019	August 6, 2019	September 2, 2019	September 10, 2019	September 22, 2019	September 25, 2019	October 1, 2019	October 21, 2019	November 2, 2019	March 3, 2020	August 3, 2020	December 14, 2020	July 13, 2021
TB	0–1.2 mg/dL	0.81	1.11	0.74	0.78	0.7	0.87	0.54	0.61	0.91	1.22^a^	0.54	0.97	1.17	0.62
DB	0–0.3 mg/dL	0.52^a^	0.79^a^	0.39^a^	0.36^a^	0.3	0.42^a^	0.28	0.19	0.42^a^	1.01^a^	0.12	0.55^a^	0.79^a^	0.36^a^
AST	5–40 U/L	29	30	46^a^	69^a^	69^a^	278^a^	449^a^	156^a^	40	43^a^	86^a^	214^a^	195^a^	54^a^
ALT	5–41 U/L	21	58^a^	82^a^	80^a^	74^a^	125^a^	263^a^	222^a^	36	36	62^a^	125^a^	77^a^	75^a^
ALP	40–129 IU/L	129	148^a^	248^a^	204^a^	237^a^	184^a^	301^a^	366^a^	252^a^	303^a^	127	139^a^	155^a^	122
Alb	3.2–4.5 g/dL	—	2.9^a^	3.9	3.9	3.7	3.3	3^a^	3.8	3.5	3.3	3.9	3.6	3^a^	3.9
Glo	2.0–3.5 g/dL	—	2.4	2.8	2.7	2.8	2.7	2.5	2.6	3.1	3.5	2.9	3.3	3.4	3.1
Mgmt	—	Baseline: day of transplant	—	—	D/C acyclovir, cotrimoxazole	—	D/C itraconazole and simvastatin	—	—	—	—	—	—	—	—

Abbreviations: Alb, albumin; ALP, alkaline phosphatase; ALT, alanine transaminase; AST, aspartate transaminase; DB, direct bilirubin; Glo, globulin; TB, total bilirubin.

^a^Outside reference range.

## Data Availability

The *Rocahepevirus* genome described in this study can be found in GenBank at the following accession: PX021458. All R and Python scripts utilized in this study can be obtained, with reasonable request, from the authors.
